# ST-Elevation Myocardial Infarction Systems of Care in Africa: A Scoping Review

**DOI:** 10.5334/gh.1524

**Published:** 2026-02-17

**Authors:** Albertus Johannes Pool, Pierre Christo Smit, Helen Slabber, Willem Stassen

**Affiliations:** 1 Division of Emergency Medicine, University of Cape Town, Cape Town, South Africa; 2 Department of Emergency Medical Care, University of Johannesburg, Johannesburg, South Africa

**Keywords:** ST-Elevation Myocardial Infarction, Healthcare Systems, Scoping Review, Africa

## Abstract

**Background:**

ST-elevation myocardial infarction (STEMI) is a life-threatening, time-sensitive emergency. Cardiovascular diseases, including STEMI, are increasing on the African continent. Improving optimal outcomes for these patients requires a system-wide approach as the existing literature is unclear.

**Objectives:**

To describe and summarise the African literature on STEMI Systems of Care (STEMI SOC).

**Methods:**

This scoping review was designed following the PRISMA-ScR guidelines. An a priori search strategy was applied to EbscoHost, PubMed, and Google Scholar databases.

**Results:**

A total of 671 articles were identified. Following the exclusion of 619 articles, 52 articles were eligible for inclusion. STEMI patients in Africa are generally younger than their Western counterparts, present late to healthcare facilities, have insufficient healthcare insurance, and are non-adherent to discharge medication. Emergency medical services are lacking, there is a shortage of percutaneous coronary intervention (PCI) facilities, and emergency departments are disorganised. STEMI reperfusion times are delayed, data collection and quality assurance initiatives are inadequate, and STEMI referral networks and registries are underdeveloped. In addition, there is a deficiency of ECG and telemetry, a shortage of healthcare workers, a lack of adherence to guideline-recommended therapy, and a perceived hesitancy of medical personnel to administer fibrinolytics. These findings suggest a need for more clinical education.

**Conclusion:**

A myriad of barriers, as well as potential facilitators in the implementation of these networks, have been reported in this scoping review. The coordination and introduction of a STEMI SOC in African settings potentially holds great advantages, as has been witnessed in other low- and middle-income countries (LMICs) and high-income countries (HICs).

## Introduction

Ischaemic heart diseases (IHDs) are the leading cause of death and disability globally (1). IHD accounts for more than one out of every ten premature deaths caused by non-communicable diseases (2).

High-income countries (HICs) have seen a decrease in cardiovascular disease (CVD) mortality, whereas low- and middle-income countries (LMICs) are experiencing a rise (3,4). It is estimated that 80% of CVD deaths now occur in LMICs, consequently impacting the younger working-age population, with significant direct and indirect economic consequences (5). This phenomenon is due to better care and prevention in HICs in comparison to urbanisation, lifestyle changes, population growth, high HIV rates, ageing, and changing health epidemiology in LMICs (4). Furthermore, it is predicted that the incidence of CVD will double in Sub-Saharan Africa and is projected by the World Health Organization to overtake communicable, maternal, perinatal, and nutritional diseases as the leading cause of death within the next two decades (3,6).

ST-elevation myocardial infarction (STEMI) is an urgent manifestation of IHD. Primary percutaneous coronary intervention (PPCI) is the treatment modality of choice if it can be performed within 120 minutes of first medical contact (FMC) (1). However, if PPCI cannot be performed within 120 minutes of FMC, fibrinolysis is an alternative option (1). Fibrinolysis may be a viable option in LMICs with limited resources (5).

STEMI Systems of Care (STEMI SOC) organise the healthcare system’s approach to STEMI to decrease time delays and improve outcomes (1,5,7). In several systems, including LMICs, the introduction of regional STEMI SOCs has had positive results in reducing treatment delays as can be seen in India, Brazil, and rural America (8,9,10,11).

In Africa, a dire shortage of PCI facilities, significant delays to reperfusion, and poor access to PCI based on geography and socio-economic status are also observed (6,12,13,14). The implementation of well-organised STEMI SOCs, integrating prehospital and in-hospital STEMI management, can help decrease these delays (9).

## Aim

The aim of the study was to describe and summarise the body of literature pertaining to STEMI SOC, as well as the barriers to and solutions for STEMI SOC implementation in the African context.

## Methods

This scoping review was designed following the Preferred Reporting Items for Systematic reviews and Meta-Analyses extension for Scoping Reviews (PRISMA-ScR) guidelines (15).

### Search strategy

The search strategy consisted of three elements:

1. STEMI Systems of Care
2. STEMI
3. Africa

These three elements were combined to compile a comprehensive search strategy to answer the research question. Searches were conducted in EbscoHost, Medline via PubMed, and Google Scholar databases, respectively, on 17, 18, and 19 October 2023. For Google Scholar, the first 10 pages were reviewed. The search strategy was refined in consultation with a librarian to improve its appropriateness and accuracy, and the full strategy is provided in Appendix A. An updated search was performed on 7 April 2025 to include more recent literature.

#### Inclusion/exclusion criteria

All study types that collected primary data or analysed existing data sets, as well as conference abstracts and reports, were included. Literature published in any language was initially considered, although the search strings were in English. Only studies published between 1 August 2003 and 31 March 2025 were included in the study. Studies for which the full text was not available, or that were not in English, were excluded.

Duplicate studies were manually eliminated by AJP. AJP and PCS independently assessed the studies for eligibility, first by reviewing the title, then the abstract, and thereafter the full text. The reference lists of the included full-text articles were examined in the same manner. Any discrepancies were resolved by WS, acting as the supervisor and independent reviewer. Screening and data management were conducted using Rayyan (16).

### Data extraction and analysis

Data were extracted by AJP from the included literature into an Excel spreadsheet (Microsoft Corporation, Redmond, WA, USA), which summarised the title, country of origin, purpose, sample, methodology, and information relating to STEMI SOC. After extraction, the data were subjected to descriptive analysis to generate a summary of the main themes identified in the literature. A formal risk-of-bias assessment was not undertaken in this scoping review, as the objective was to broadly map and synthesise all available evidence, regardless of quality.

## Ethical considerations

As this study did not include human participants or any patient-level data, an exemption from ethical review was obtained from the Human Research Ethics Committee of the University of Cape Town (HREC reference 063/2024).

## Results

### Overview

**Figure 1:**
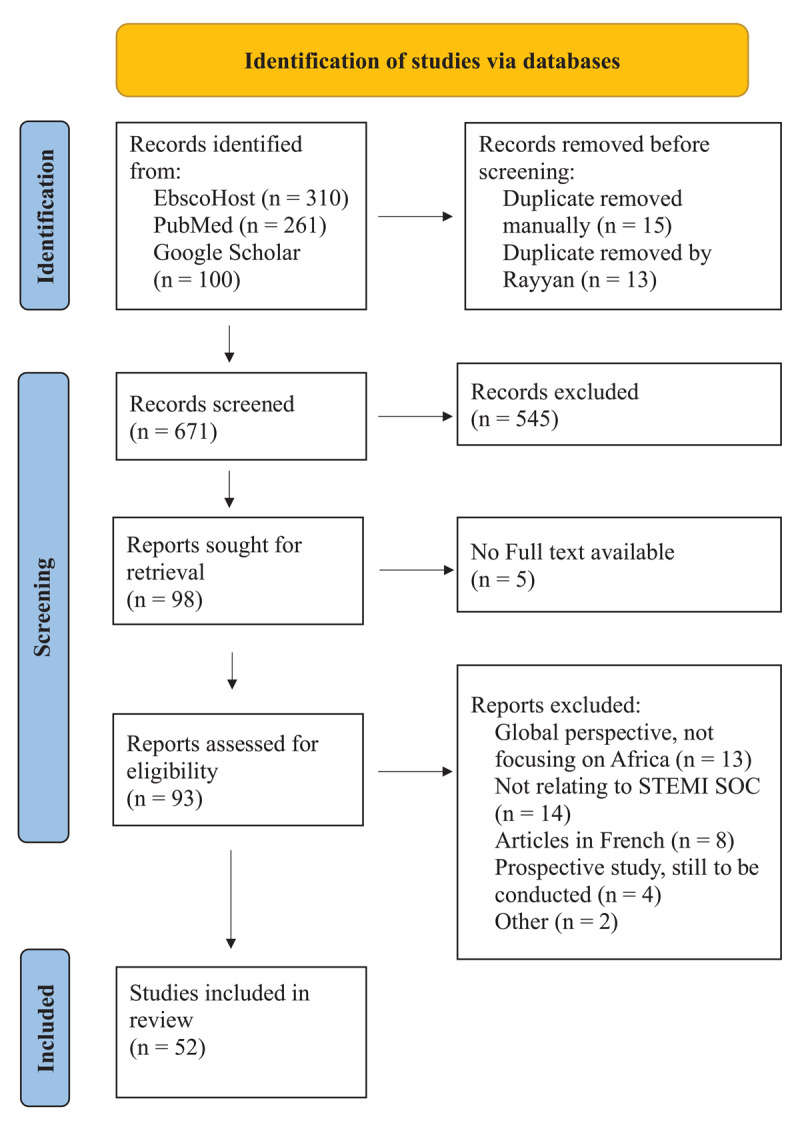
PRISMA flow diagram of database searches conducted.

A total of 671 articles were identified through the database search. After screening, 52 articles were deemed eligible for inclusion. Figure 1 outlines the PRISMA flow diagram for article selection.

**Figure 2:**
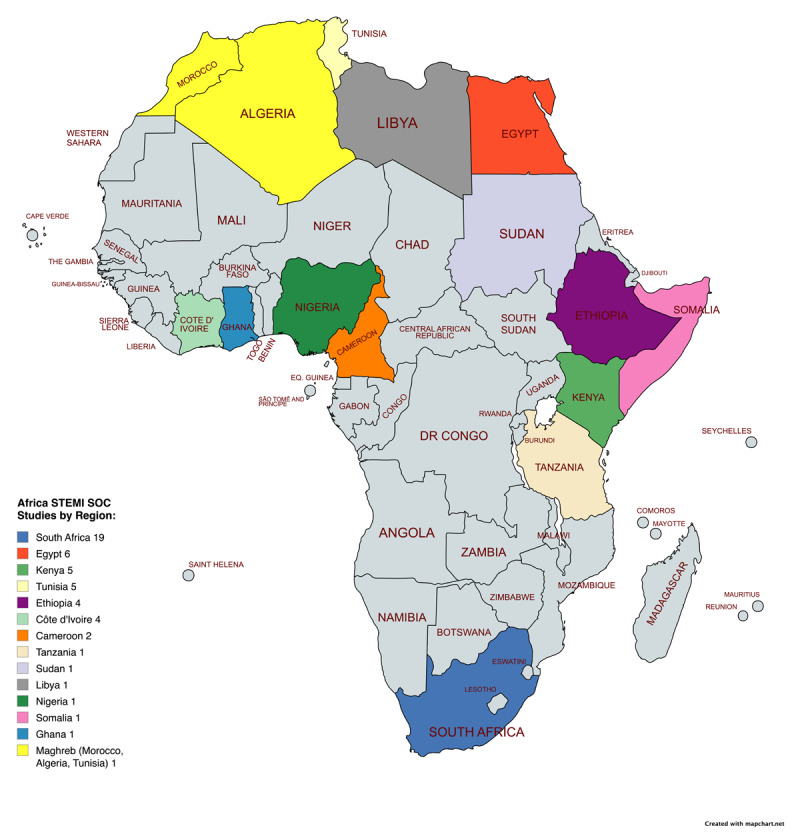
Article’s originating countries.

The included articles originated from studies conducted in South Africa (37%), Egypt (12%), Kenya (10%), Tunisia (10%), Ethiopia (8%), Côte d’Ivoire (8%), and Cameroon (4%). In addition, one study each was identified from Ghana, Sudan, Tanzania, Libya, Nigeria, Somalia, and the combined Maghreb region. Figure 2 illustrates the countries that were represented.

Most of the studies were facility based (69%), followed by studies conducted both in- and out-of-hospital (14%), and out-of-hospital (6%) studies. The majority of studies used a quantitative approach (81%), followed by qualitative (8%) and mixed-methods (6%) designs. Retrospective (40%) and prospective observational studies (37.5%) accounted for the majority of quantitative studies. Qualitative studies were conducted through individual (50%) and group interviews (50%).

Results were grouped into seven themes. These were patient-related challenges, healthcare funding challenges, prehospital challenges, policy and quality assurance, healthcare facility challenges, technology gaps, and healthcare worker challenges. The articles are presented within this thematic framework in the extraction sheet (Appendix B).

### Patient-related challenges

A common theme reported in every country was delayed presentation of acute coronary syndrome (ACS) to healthcare facilities. This is one of the main barriers to timely STEMI care in Africa with median symptom-onset-to-presentation times reported ranging from 3.5 hours in South Africa, 5.3 hours in Egypt, 7 hours in the Maghreb region, 12.5 hours in Kenya, 20 hours in Côte d’Ivoire, while a large proportion of patients in Sudan arrived after 24 hours. Ethiopian researchers reported patients arriving after 26 hours, with nearly two-thirds of patients presenting with heart failure symptoms. Similar delays were reported in countries such as Senegal, Tunisia, Libya, and Nigeria (12,13,14,17,18,19,20,21,22,23,24,25,26,27,28,29,30,31,32,33,34,35,36,37,38,39,40,41,42).

Reasons for these delays include patients’ inability to recognise the signs and symptoms of STEMI (13,21,22,24,26,27,35,38,43,44,45,46), education levels, and socio-economic status (20,26,44,47). In Nigeria, STEMI patients in lower socio-economic categories were 3.1 times more likely to die than their high-income counterparts (20). Egyptian investigators reported a reduction in FMC-to-balloon time from 230 minutes in 2011 to 60 minutes in 2016–2017, attributed in part to a social media campaign, TV advertisements, press releases, and public sessions (48). Policymakers have also advocated for the inclusion of family members in education programs, as this increased the likelihood of patients acting early (26). Other challenges include patients’ reluctance to seek help (24,25), long travel distances (21,25,37,44), hospital resource limitations (22,35), emergency medical services (EMS) resource limitations (22,44), self-medication (24), and prolonged hospital triage times (22).

Other themes identified in the literature include patient’s long-term non-adherence to discharge medications after an ACS event (22,46,49), younger age at presentations among HIV-positive STEMI patients (50,51), and delayed treatment times that worsen outcomes in both the elderly and women (21,31,52). A significant and universal finding was that STEMI patients in Africa are generally younger than those in Western countries (14,20,22,23,24,28,29,33,34,35,38,42,44,48,49,50,53,54) (Table 1).

**Table 1:** Patient-related challenges.


BARRIER	COUNTRY	COMMENTS	POTENTIAL SOLUTION

Delayed patient presentation	RSA, Egypt, Tunisia, Maghreb region, Kenya, Senegal, Ethiopia, Côte d’Ivoire, Libia, Nigeria, Sudan	Lack of ACS symptom recognition Education level Socio-economic status Reluctance to seek help	Effective education campaigns for patients and healthcare workers Awareness of early STEMI management Build trust with patient and families

Long travel distance	Egypt, Sudan, Ethiopia		Telemedicine Task shifting Inclusion of PHCs and small clinics as spokes

Non-adherence to discharge medications	RSA, Tanzania		Education Policy guidelines

Younger age STEMI and HIV presentation	RSA		Early management of risk factors

Delayed treatment times in elderly and women	Tunisia, Sudan		Education

Younger age STEMI presentation	RSA, Egypt, Maghreb region, Kenya, Ethiopia, Côte d’Ivoire, Nigeria, Tunisia		Early management of risk factors

**FACILITATOR**	**COUNTRY**	**COMMENTS**	**RESULTS**

Education campaigns	Egypt	Education campaigns focusing on social media, TV advertisements, press releases, and public sessions, including the education of relatives.	Decrease in FMC-to-balloon time.


Abbreviations: ACS, acute coronary syndrome; FMC, first medical contact; Maghreb, combined Tunisia, Morocco, and Algeria; PHC, primary healthcare centre; RSA, Republic of South Africa; STEMI, ST-elevation myocardial infarction.

### Healthcare funding challenges

African countries present a diverse range of challenges related to funding. According to Tantchou et al., health insurance practically does not exist in Cameroon, leaving the families indebted (55). In Côte d›Ivoire, the cost of the ‘cheap’ streptokinase reaches USD263, and in Nigeria the drug is not a prioritised in standard guidelines and is hardly affordable (20,37).

A lack of health insurance is raised as barriers to effective STEMI care in a few countries (20,41,42,44). This leads to non-testing of cardiac biomarkers, using bare metal stents (BMS) instead of drug-eluding stents (DES), inaccessibility to P2Y12 inhibitors, poor guideline adherence, non-utilisation of adjunct anti-platelet therapy, and lack of prehospital thrombolysis (20,38,41,42,56). In the Maghreb region, 62% of patients had government health insurance (33), and in Egypt, up to 93% of patients were covered for STEMI treatment expenses (25,29).

In South Africa, the private sector owns the majority of percutaneous coronary intervention (PCI) facilities, which can therefore be utilised by only 18.1% of the population unless an upfront payment of up to USD3,500 is made (57). Stassen et al., Tickley et al., and Meel et al. report delays and denial of thrombolysis in private hospitals due to requests for upfront payment (13,18,24). Snyders et al. describe delays caused by health insurers mandating transfers from one PCI facility to another (45).

A cost-effectiveness analysis conducted in Ethiopia found that an integrated package consisting of aspirin, ACE-inhibitor, beta-blocker, and streptokinase yielded significant cost-benefit compared to a highly skilled intervention consisting of PCI, aspirin, and clopidogrel. Their results indicated the need to reprioritise basic pharmacologic regimens for AMI treatment in resource-constrained settings rather than investing in high-cost interventions like PCI (40) (Table 2).

**Table 2:** Healthcare funding challenges.


BARRIER	COUNTRY	COMMENTS	POTENTIAL SOLUTION

Lack of health insurance	Cameroon, Côte d’Ivoire, Nigeria, Ethiopia, RSA		Government funding for STEMI care Bundling cost of STEMI care Education about importance of treatment costs and insurance Small amount to be paid by patient

Private sector owned facilities	RSA	Can only be availed by insured patients or after significant upfront payments. Causes delays and denial of thrombolysis	Government social funding for STEMI care to those below poverty line Public private partnerships

**FACILITATOR**	**COUNTRY**	**COMMENTS**	**RESULTS**

Health insurance available	Egypt, Maghreb region		Increase access to STEMI care

Cost-effective analysis	Ethiopia	An integrated package consisting of aspirin, ACE-inhibitor, beta-blocker, and streptokinase yielded significant cost-benefit compared to PPCI	Advised to reprioritise basic pharmacologic regimens for AMI treatment in resource-constrained settings rather than investing in high-cost interventions like PCI


Abbreviations: AMI, acute myocardial infarction; PPCI, primary percutaneous coronary intervention; RSA, Republic of South Africa; STEMI, ST-elevation myocardial infarction.

### Prehospital challenges

EMS transportation of STEMI patients in Africa is negligible. Usage rates are reported as 9.4% in Ethiopia, 11.7% in Nigeria, 11.9–27.7% in Tunisia, 22% in Egypt, and a wide range of 16–51% in different sectors in South Africa (12,13,20,23,24,25,31,44,54). This is due to the limited availability of EMS or an overburdened service (13,18,22,24,25,26,36,42,44). In Nigeria, the EMS was described as practically non-existent (20). Other challenges facing EMS in Africa include the lack of ECGs and regulatory barriers to prehospital fibrinolysis (14,18,45,56). Tunisia and Senegal, however, reported practising prehospital fibrinolysis in their respective prehospital settings (37,58) (Table 3).

**Table 3:** Prehospital challenges.


BARRIER	COUNTRY	COMMENTS	POTENTIAL SOLUTION

Limited/overburdened EMS	Ethiopia, Nigeria, Tunisia, Egypt, RSA, Côte d’Ivoire, Tanzania		Government funding to increase availability of EMS Trained prehospital providers Transfer policies in place between PCI and non-PCI facilities Availability of telemedicine Availability of bolus thrombolytics

**FACILITATOR**	**COUNTRY**	**COMMENTS**	**RESULTS**

Accessibility of prehospital fibrinolysis	Tunisia, Senegal		Shorten delay to fibrinolysis


Abbreviations: EMS, emergency medical services; PCI, percutaneous coronary intervention; RSA, Republic of South Africa.

### Healthcare facility challenges and referral

Healthcare facilities in Africa are plagued by disorganised emergency departments (EDs), inadequate infrastructure, and resource limitations (13,14,23,41,42,53). These contribute to significant in-hospital delays in STEMI reperfusion and medication shortages. The ACCESS registry in the Maghreb region reported that 42% of eligible STEMI patients received no reperfusion therapy within 24 hours and 30% of patients underwent fibrinolysis, with vast majority (92%) receiving streptokinase. This was due to cost and resource limitations. Only 27% of eligible STEMI patients received PPCI (33).

None of the STEMI patients admitted to two tertiary hospitals in Ethiopia received thrombolytic medication, and only 7.2% of patients underwent PCI. This shortfall in STEMI management was due to lack of medication and limited PCI facilities (44). These limitations contributed to longer in-hospital stays and guideline non-adherence, resulting in very few patients receiving morphine (12.9%) and nitrates (35.5%) in the ED (38).

Côte d’Ivoire reported fibrinolysis being performed in 11.3–13% of patients within 12 hours of symptom onset, with 19.8–21.3% of patients receiving primary PCI. Moreover, the most commonly used stents were BMS (41,42). Feedback from the Afri-Cardio conference indicated a severe shortage of PCI facilities, with only five cath-labs available in the 10 participating countries. In Abidjan and Dakar, new facilities have been established, and thrombolysis rates increased from 11.3% to 31% in less than a decade (37).

A public PCI facility in Kenya reported that 5% of eligible STEMI patients received thrombolysis and 12% received PCI (53). Data from a private facility in Kenya demonstrated that 53.1–55% of patients received thrombolysis and 13–18% received primary PCI. Door-to-needle (D2 N) times were 47–49 minutes, and door-to-balloon (D2B) times were reported as 84 and 137 minutes, respectively. The shortage of PCI facilities and cost implications were contributing factors (35,36). Due to the low volume of primary PCIs conducted in the region, maintaining a 24-hour on-site team is not yet cost-effective (35). Likewise, Libya, Somalia, and Nigeria all reported delayed reperfusion rates within their systems (19,20,59).

In South Africa, there is currently one PCI facility for every 887,096 people, of which the majority are in the private sector, concentrated in major cities, and unevenly distributed with some high-population-density areas lacking adequate facilities (57,60,61,62). Earlier ACCESS registry data from South Africa indicated that 18% of patients received thrombolysis on admission, with streptokinase used in 54.5% and tenecteplase in 30.3% of cases. PCI was performed within 24 hours of hospitalisation in 61.3% of cases (14). A pilot study in South Africa concluded that there were significant delays with fibrinolysis administered within 30 minutes in 50% of direct access (DA) patients and 20% of inter-facility transfer (IFT) patients. Primary PCI within 60 minutes of FMC was achieved in only 13% of DA patients and in none of the IFT patients (45). Several observational studies in South Africa report on reperfusion times. Meel et al. reported that only 37% of eligible patients received fibrinolytic therapy, of which only 3% received it within one hour. Median D2 N times were reported as 60 minutes for those receiving the agent in the ED and 85 minutes for those receiving it in the coronary care unit (CCU). Other median D2 N times were reported to range from 54 to 183 minutes. Reasons for these delays included shortcomings in patient flow, junior doctors consulting senior doctors, lack of coordinated systems of care, busy health facilities, and unavailability of thrombolytics (12,23,24). Fortunately, Chetty et al. reported D2 N times of 43 minutes, citing the availability of trained physicians and expert consultation decreasing perfusion times (63).

Two authors reported on a hub-and-spoke model in Cape Town, South Africa. Patients present to their respective primary or secondary healthcare facility, where those with STEMI receive thrombolytics, with referral to tertiary institutions for failed thrombolysis. Within this STEMI SOC, angiography is also conducted at PCI facilities (22,50). However, these systems remain challenged by prolonged triage times, limited resources, high patient burden, and insufficient ECG diagnostic proficiency. This is evidenced by only 39.6% of STEMI patients receiving thrombolysis, with median times from diagnosis to fibrinolysis of 67 minutes (22,49).

Earlier data from Tunisia reported median diagnosis-to-reperfusion times of 46 minutes for direct presenters and 110 minutes for transferred patients. Causes of delays were inter-department decisions (off-site cardiologist vs general practitioner in the ED) and IFTs (58). More recent results from the FAST-MI Tunisia registry, which captured data from 72.2% of Tunisian public hospitals, indicate a substantial increase in reperfusion rates. Fibrinolysis was administered in 31.8% of patients, of which 27.7% occurred in the prehospital setting, and primary PCI was performed in 30% of patients. Median times from symptom onset to fibrinolysis were 180 minutes, and 360 minutes for primary PCI. This was in part due to greater adherence to recommendations and better organisation among STEMI treatment stakeholders. Some identified limitations included low levels of prehospital fibrinolysis, limited direct PCI admission, and poorly structured healthcare networks in regional hospitals (54).

Egyptian authors were the first to describe the STEMI Stent for Life initiative, which was launched in 2011. A registry was created after meetings to resolve previously identified barriers. Following the meetings, a press conference, patient awareness campaigns, and physician education meetings were held. The cardiology board met with the Minister of Health and subsequently received support for the initiative through covering the PCI procedure expenses, initiating a STEMI protocol, EMS training and acquiring new ECGs, rapid ECG transmission, and supporting public awareness campaigns (29). They reported an improvement of primary PCI rates along with median D2B times. BMS were still used in 80.7% of cases compared to 19.3% receiving DES (29).

Also in Egypt, a multicentre registry study evaluating their STEMI SOC from 2014 to 2017 was conducted. An increase in immediate transfers to PCI facilities, increased number of PCIs, a decrease in fibrinolysis, and a significant decrease in D2B times from 60 to 50 minutes were reported (48). The authors credited their education campaigns for these improvements. A hub-and-spoke model in Northern Cairo was established with one main PCI-capable hospital as the hub, and three referring spoke hospitals. WhatsApp® was used as a digital communication platform to share ECG and relevant patient data. This allowed for swift diagnosis and urgent transfer by EMS to the hub hospital for primary PCI. A pharmacoinvasive (PI) approach ensued in cases of expected delays. A STEMI protocol, along with flowcharts, were applied by the hub, spokes, and EMS. Training sessions were held to demonstrate the value of early reperfusion in STEMI patients. Results were observed and reported on pre- and post-implementation of their STEMI SOC. Median time from symptom onset to FMC was unchanged between the two groups; however, D2B times were reduced from 54.3 to 44.1 minutes (D2 N times remained unchanged). The use of fibrinolysis decreased significantly from 36.4% to 7.5%, while primary PCI increased from 59.8% to 77.1%. Both median CCU and total hospital stay days decreased after the STEMI SOC implementation. In-hospital mortality improved from 6.4% to 2.8%. The authors concluded their STEMI SOC was feasible and improved patient outcomes (28). An Egyptian qualitative survey identified limited resources, lack of trained interventional cardiologists, insufficient CCU beds, and lack of regional STEMI networks and policies as the main barriers in the management of STEMI patients (25) (Table 4).

**Table 4:** Healthcare facility challenges and referral.


BARRIER	COUNTRY	COMMENTS	POTENTIAL SOLUTION

Inefficient healthcare facilities	RSA, Côte d’Ivoire, Kenya, Maghreb region, Libya, Somalia, Nigeria, Egypt	Delays in STEMI treatment Ineffective patient flow Junior healthcare providers Uncoordinated systems of care	Government funding ECG analysis campaigns Implement STEMI Systems of Care Geographic mapping Feedback and quality assurance initiatives Pharmacoinvasive approach

Lack of resources	Ethiopia, Côte d’Ivoire, Kenya, RSA, Egypt	Lack of medications Utilisation of BMS Lack of CCU beds	Government funding Better use of health insurance scheme Lobbying to government that organisation of care is cost-effective and essential for resource optimisation Focus on set-up of 24/7 hub, and then spokes with access to fibrinolytics

**FACILITATOR**	**COUNTRY**	**COMMENTS**	**RESULTS**

Establishment of new PCI facilities	Côte d’Ivoire, Senegal		Thrombolysis rates increased from 11.3% to 31%

Availability of trained physicians and expert consultation	RSA		Decreased reperfusion times

Hub-and-spoke model	RSA	STEMI patients receive thrombolytics at spoke hospital, and further referral to tertiary institutions for failed thrombolysis if required	However, these systems are still troubled by prolonged triage times, limited resources, a high patient burden, and insufficient ECG diagnostic proficiency

Greater adherence to recommendations and better organisation of STEMI treatment stakeholders	Tunisia		A substantial increase in reperfusion rates

STEMI stent for life initiative	Egypt		An improvement of primary PCI rates along with median D2B times

Education campaigns	Egypt		Increase in immediate transfers to PCI facilities, increased number of PCIs, a decrease in fibrinolysis, and a significant decrease in D2B times

Hub-and-spoke model	Egypt		D2B times were reduced, the use of fibrinolysis decreased, primary PCI increased, both median CCU and total hospital stay days decreased, In-hospital mortality improved from 6.4% to 2.8%.


Abbreviations: BMS, bare metal stents; CCU, coronary care unit; D2B, door-to-balloon; Maghreb region, Morocco, Tunisia, Algeria; PCI, percutaneous coronary intervention; RSA, Republic of South Africa; STEMI, ST-elevation myocardial infarction.

### Policies, legislation, and quality assurance

Several studies reported on a lack of guideline adherences. Discharge medication post-STEMI has been shown to be effective in reducing complications such as re-infarction and death; these include dual-antiplatelet medications (aspirin and clopidogrel), beta-blockers, ACE-inhibitors, and statins (31,38,53). Ethiopian and Kenyan data reveal 61.1% and 56% compliance rates, while Nigerian and Tunisian data indicated a need to increase adherence to international guidelines (20,31,38,44,53). South African authors revealed marginally better adherence with 83.6% of patients receiving appropriate secondary prevention prescriptions (49).

Various authors identified the need to implement policies between receiving and referring hospitals. Tickley et al. commented that there was no STEMI network in place in their tertiary institution in Johannesburg (24). Shaheen et al. recommend STEMI management protocols to be in place, to encourage direct cath-lab admission, repatriation policies post-STEMI management, and implementation of medical codes for post-thrombolysis (25). The authors further reported that only 21% of PCI centres and 8% of non-PCI centres had STEMI management protocols (25). By implementing these recommendations, along with the PI approach, systems were able to decrease reperfusion times, increase primary PCI, decrease fibrinolysis, and improve mortality in Egypt (28,48). DIDO times were reported as 40 minutes in Egypt, one hour in Tunisia, and up to eight hours in South Africa (13,26,28,58). In South Africa and Kenya, long delays and disorganised ECG interpretation flows were noted (12,24,53).

The introduction of STEMI registries was reported in Tunisia, South Africa, and Nigeria (14,20,54). These registries stimulated improvement in ACS care and outcomes in Nigeria and increased PCI access in South Africa (14,20). In addition, they aided in data collection to assess time trends, current status and areas for improvement, and patient characteristics in Kenya and Egypt (36,48,53). Shaheen et al. reported on quality control indicators for STEMI diagnosis and management being variably implemented in PCI, and less so in non-PCI facilities (25) (Table 5).

**Table 5:** Policies, legislation, and quality insurance.


BARRIER	COUNTRY	COMMENTS	POTENTIAL SOLUTION

Non-compliance to discharge medication	Ethiopia, Kenya, Nigeria, Tunisia		Discharge algorithms, policies Awareness campaigns

Absence of STEMI SOC	RSA, Egypt, Tunisia, Kenya		Create STEMI networks in individual communities Legislation to by-pass non-PCI/fibrinolysis capable hospitals

**FACILITATOR**	**COUNTRY**	**COMMENTS**	**RESULTS**

STEMI register introduction	Tunisia, RSA, Nigeria, Kenya		Stimulated improvement in ACS care, increased PCI, aided in data collection to assess time trends, current status, and areas for improvement


Abbreviations: ACS, acute coronary syndrome; PCI, percutaneous coronary intervention; RSA, Republic of South Africa; STEMI SOC, ST-elevation myocardial infarction Systems of Care.

### Technology gaps

Several authors commented on the lack of ECGs and technology in their pre- and in-hospital systems in Egypt, Kenya, and South Africa (13,24,25,35,36). Stassen et al. and Shaheen et al. both mentioned the use of WhatsApp® amongst STEMI management role players to send ECGs to improve STEMI care (18,28). Snyders et al. reported that paramedics were taking photos of ECGs and sending it to cardiologists for expert consultation (45). Researchers from Côte d’Ivoire reported on their hub-and-spoke telecardiology project, whereby cardiologists were able to support non-urban hospitals in early diagnosis and treatment of STEMI (64). In addition, Coetzee et al. and Stassen et al. presented the use of geospatial analysis to determine STEMI treatment and transportation pathways in South Africa (61,62) (Table 6).

**Table 6:** Technology gaps.


BARRIER	COUNTRY	COMMENTS	POTENTIAL SOLUTION

Lack of ECGs	Egypt, Kenya, RSA		Explore cheap but high-quality ECG machines like STEMI India ECG telemetry from FMC to higher centre

**FACILITATOR**	**COUNTRY**	**COMMENTS**	**RESULTS**

Use of instant messaging for telemetry purposes	RSA, Egypt	Use of WhatsApp® to send ECGs	Improve STEMI care.

Telecardiology	Côte d’Ivoire	Hub-and-spoke telecardiology project	Cardiologists were able to support non-urban hospitals in early diagnosis and treatment of STEMI

GIS	RSA		Use of geospatial analysis to determine STEMI treatment and transportation pathways


Abbreviations: FMC, first medical contact; GIS, geographic information system; RSA, Republic of South Africa; STEMI, ST-elevation myocardial infarction.

### Healthcare worker challenges

Many African countries raised the concern of a shortage of medical staff. Cameroon, by example, only has one doctor per 12,500 people; Egypt, Kenya, South Africa, and Ethiopia all reported a shortage of trained interventional cardiologists, nurses, and technicians (25,36,44,55).

Kenyan authors referred to poorly exposed healthcare workers, failing to diagnose STEMI on ECG (36). This was echoed in South Africa as well, where ECG misdiagnosis delayed fibrinolytic therapy (13). ECG misdiagnosis was reported as 10%, 16%, and up to 29.2% (22,23,24). A recurring theme in South Africa was a perceived hesitancy towards the administration of thrombolytics (17,18,24,56). Furthermore, there was a need for having expert opinion readily available to assist in the diagnosis of STEMI on ECG (13,58,63).

Egyptian and Tunisian authors report in their respective STEMI SOC on training within their networks. Mohamed et al. described continuous medical education campaigns for more than 3,700 cardiologists, emphasising on the importance of immediate transfers. This training was subsequently expanded to prehospital providers and referral physicians (48). Shaheen et al. conducted training sessions for doctors, nurses, and technicians, which included performing quality ECGs, early diagnosis and rapid referral, and sending ECGs through WhatsApp® (28). A training program was conducted in Tunisia for ED physicians to demystify the perceived risk of fibrinolysis, while another programme improved ACS management and was subsequently expanded to other regions (31,58) (Table 7).

**Table 7:** Healthcare worker challenges.


BARRIER	COUNTRY	COMMENTS	POTENTIAL SOLUTION

Healthcare worker shortage	Cameroon, Egypt, RSA, Kenya, Ethiopia	Shortage of trained interventional cardiologists, nurses, and technicians.	Task shifting HCW incentives

Hesitancy in ECG diagnosis and thrombolytic administration	Kenya, RSA	Delayed fibrinolytic therapy.	HCW training campaigns

**FACILITATOR**	**COUNTRY**	**COMMENTS**	**RESULTS**

Healthcare worker training	Egypt, Tunisia	CME campaigns	Improved ACS management


Abbreviations: ACS, acute coronary syndrome; CME, continuous mandatory education; HCW, healthcare worker; RSA, Republic of South Africa.

## Discussion

This scoping review set out to describe and summarise African literature on STEMI SOC, as well as barriers and solutions to STEMI SOC implementation. Overall STEMI SOC literature is scarce in Africa. Out of the 52 included articles, only Egypt, South Africa, and Tunisia made reference to formal STEMI SOC within their countries (22,27,28,29,48,49,50,54). Of these three, Egypt has shown their STEMI SOC implementation to be feasible, improves patient outcomes, and increases access to PPCI (28,48). The seven themes identified were largely echoed in reviews by Mehta et al. and Nascimento et al. on STEMI SOC in LMICs and India (65,66).

Time to treatment is critical to preserve myocardium. Any delay will impact myocardium salvage (1,5). Delays in symptom onset to FMC was also witnessed in other LMICs like India and Brazil (10,67). Several recommendations have been made regarding focus on public education of signs and symptoms of STEMI, early diagnosis, early reperfusion, and the importance of using EMS. Chandrashekar et al. recommend public service announcements and educational campaigns educating the public through social media, entertainment channels, community theatres, and mobile messaging platforms (5). For educational messages to be effective, they need to be targeted at the right group, written in simple language, be comprehensible to all levels of education, and be clear and concise. For bigger impact, it has been suggested to build this message into narratives of movies and television shows (65). Specific to Africa, Asamoah et al. suggest enlisting the assistance of cultural and religious leaders, as African communities have a strong sense of family and community (68). Countries should explore these recommendations within their respective localities to target the intended group and decrease patient delays in presentation.

Reasons for the younger age of STEMI patients reported in this review are diverse and regional, with causes attributed due to higher prevalence, and at times undertreatment of risk factors, insufficient prevention programmes, urbanisation and lifestyle changes, shorter lifespan, HIV prevalence, non-adherence to chronic medication, recreational drug use, and higher salt intake (19,20,22,23,28,29,31,33,34,36,41,42,48,50,51,53,54,63). African countries should focus on their specific burdens and implement proven strategies towards primary prevention.

Substantial progress can be made with government buy-in, as was seen in Egypt (27,29). To improve SOC, governments, NGOs, and other stakeholders should find ways to cover healthcare costs for STEMI patients. Funding should be directed to cover all patients, regardless of their ability to pay. Policymakers should negotiate costs and minimal pricing for drugs and devices through bulk purchasing (5). This is essential given that out-of-pocket expenses is a major contributor towards patient-related delay. If any, a small co-payment could be levied to promote awareness and co-participation in their own well-being amongst patients (65). Notably, in other LMICs, investments in STEMI SOC yielded economic benefits to society, justifying governmental investment (69,70). Investments then should be prioritised to ensure equal access to ACS care. Examples of where this has been successfully introduced in LMICs include India’s Tamil Nadu Social Insurance Scheme and the Brazilian United Health Service where free STEMI care has been introduced (10,71).

The low rate of EMS usage reported herein might be explained given that only 8.7% of the African population is covered by a prehospital service (72). EMS serves as the entry point to STEMI care and is instrumental in improving outcomes as they have the potential to detect STEMI early on, direct patients to the most appropriate facility, or initiate prehospital fibrinolysis. Investing in EMS infrastructure, ECG interpretation training, ECG telemetry, and interagency communication between EMS and PCI facilities are recommended (1,5,65,73). Several systems in HICs and LMICs report on their successes with prehospital 12-lead ECGs, telemedicine support, and direct PCI activations (11,74,75,76,77,78). A challenge, especially in Africa with its limited resources, would be false prehospital activations, which may result in excessive resource waste. This can be reduced through training, ECG transmission, clear algorithms, and utilising advanced technologies like artificial neural networks (79,80,81).

Hub-and-spoke models with a PI approach are feasible strategies in Africa in a bid to overcome limited resources. This was demonstrated in India where its implementation increased rates in both the PI strategy and PPCI (82).

The STEMI-India model showed that a dedicated team ensured collaboration amongst hospitals, EMS, and insurance agencies (5,83). This has further shown to reduce FMC to device times by reducing interhospital delays and expediting interhospital transfers (84,85,86,87,88,89). A STEMI register should be created, ideally be securely cloud based, provide real-time feedback, and be quality controlled. Furthermore, funding should allow for time allocation to staff for data collection and stakeholders for data analysis. Countries should aim to build these live registries, as seen in STEMI India, AHA’s Mission Lifeline, Brazil’s RESSIST registry, and others (5,11,82). Some STEMI registries on the continent do however exist, but with variable implementation successes, data capturing, and monitoring of outcomes amongst the different countries (14,20,54).

Recommendations are that a 12-lead ECG should be performed within 10 minutes of FMC (1,5). Government should coordinate with technology companies for innovation in supplying low-cost 12-lead ECGs. This helps in overcoming manpower and infrastructure constraints. As seen in India, a low-cost 12-lead ECG device was developed, capable of ECG transmission, vital sign monitoring, and data storage to assist in analysis and quality improvement purposes. This device can be used in the patient’s home, in an ambulance, or any hospital facility (71,83).

Clinicians should be well versed with the diagnosis of STEMI and the importance of early reperfusion (1). This can be achieved through low cost or free continuous medical education programs that are offered periodically to spoke hospital staff (83). As witnessed in Tunisia and Egypt, these training sessions achieved the wanted outcomes and improved access to STEMI reperfusion (28,31,48,58). Adequate training may allow for task shifting to EMS, which can be supported by telemedicine, while quality assurance, data collection, and management can be shifted towards administrative staff (66).

## Policy and practical implications

While common barriers to STEMI care exist across the continent, heterogeneity in culture, geography, and baseline health system development and organisation necessitates the development and testing of pragmatic, context-specific solutions. A practical first step involves conducting a pre-implementation needs assessment and implementing mechanisms of data collection to inform future plans. Lack of PCI infrastructure makes a central hub-and-spoke model to STEMI referral attractive, but where these are wholly unavailable a management strategy focusing on widespread thrombolytic availability seems wise. Regardless of the approach, it should be implemented while simultaneously strengthening EMS, including emergency medical dispatch and ambulance provider training. Leveraging ECG telemetry to support diagnosis and referral decision-making is key to overcoming inequity in care for underserved communities. ECG telemetry should be able to transmit through mobile applications, fixed phones, or the internet.

Furthermore, lobby groups and stakeholders should inform governments that STEMI SOCs are cost-effective, reduce mortality, and promote equitable access to healthcare resources (5,66). Lastly, communities should be engaged to ensure that delays in recognition and care-seeking, as well as poor treatment adherence may be addressed.

## Limitations

It is possible that some relevant studies on STEMI SOC in Africa could have been missed during the searches of the different databases. It is also possible that some viable literature could have been omitted due to being published in a different language (eight studies were excluded as they were in French). Nevertheless, the search string was employed in the appropriate databases, piloted, and tested with the assistance from a librarian skilled in search strategies, meeting the recommendations for an optimal search strategy. Furthermore, as a large part of this review was based on observational studies with their inherent risks of bias, confounding, and limited external validity, results and recommendations should be applied with caution. Other limitations were the lack of data from most of African continent and an over-representation of some systems. As such, this review cannot account for the diverse nature healthcare systems across the continent.

## Conclusion

The literature on STEMI SOC in Africa is scarce with only Egypt, Tunisia, and South Africa reporting comprehensive information on their systems. The coordination and introduction of a STEMI SOC in Africa potentially holds great advantages as has been witnessed in other LMICs and HICs. A myriad of barriers have been reported in this review, as well as potential facilitators in the implementation of these systems. Each setting will be unique and will require interagency liaison, government support, infrastructure investment, legislation, and quality assurance programs to ensure its success. Role players should endeavour approaching other LMICs and HICs, to learn from their lessons and processes—a few of which were highlighted. Ultimately, STEMI SOC has shown to be cost-effective, reduce treatment delays, allow more people to access PCI, and ultimately reduce morbidity and mortality. More research is needed in the African context, with a focus on mapping the current state of the STEMI burden and management in each locality, establishing STEMI registers, investigating the impact of technology, and exploring the possible implementation of STEMI referral networks within individual communities along with feedback and quality assurance initiatives.

## Additional Files

The additional files for this article can be found as follows:

10.5334/gh.1524.s1 Appendix A. Search Strategies.

10.5334/gh.1524.s2 Appendix B. Extraction Template.
