# Supplementary material for: ST-Elevation Myocardial Infarction Systems of Care in Africa: A Scoping Review
Source: Glob Heart. 2026 Feb 17;21(1):11. doi: 10.5334/gh.1524 (PMC12922679; doi:10.5334/gh.1524)
Supplement: Appendix A. — Search Strategies. [file gh-21-1-1524-s1.pdf]

Appendix A: Search Strategies

EBSCOhost Research Databases; Search Screen - Advanced Search; Database - Africa-Wide Information; CINAHL

("system* of care" OR "coronary care network*" OR "regional network*" OR "coronary care system*" OR "care network*" OR "community network*" OR "health network*" OR regionalisation OR regionialization OR management OR treatment) AND (STEMI OR "ST-elevation myocardial infarction" OR "heart attack*" OR "occlusion myocardial infarction" OR OMI OR "coronary occlusion" OR "ST-Segment Elevation Myocardial Infarction" OR "ST-elevated myocardial infarction" OR "st-segment elevated myocardial infarction") AND (Africa OR African OR Algeria OR Angola OR Benin OR Botswana OR "Burkina Faso" OR Burundi OR "Cabo Verde" OR Cameroon OR Cameroun OR "Canary Islands" OR "Cape Verde" OR "Central African Republic" OR Chad OR Comoros OR Congo OR "Cote d'Ivoire" OR "Democratic Republic of Congo" OR Djibouti OR Egypt OR Eritrea OR eSwatini OR Ethiopia OR Gabon OR Gambia OR Ghana OR Guinea OR Guinea- Bissau OR "Ivory Coast" OR Jamahiriya OR Kenya OR Lesotho OR Liberia OR Libya OR Madagascar OR Malawi OR Mali OR Mauritania OR Mauritius OR Mayotte OR Morocco OR Mozambique OR Namibia OR Niger OR Nigeria OR Principe OR Reunion OR Rwanda OR "Saint Helena" OR "Sao Tome" OR Senegal OR Seychelles OR "Sierra Leone" OR Somalia OR "St Helena" OR Sudan OR Swaziland OR Tanzania OR Togo OR Tunisia OR Uganda OR "Western Sahara" OR Zaire OR Zambia OR Zimbabwe)

PubMed:

((((((("ST Elevation Myocardial Infarction"[Mesh]) OR (STEMI OR ST-elevation myocardial infarction OR heart attack OR occlusion myocardial infarction OR OMI OR coronary occlusion OR ST-Segment Elevation Myocardial Infarction OR ST-elevated myocardial infarction OR st-segment elevated myocardial infarction)) OR ("Myocardial Infarction"[Mesh])) OR ("Coronary Occlusion"[Mesh])) AND (("Community Networks"[Mesh]) OR ("systems of care" OR "system of care" OR coronary care network OR regional network OR coronary care networks OR regional networks OR coronary care system OR coronary care systems OR care network OR care networks OR community network OR community networks OR health network OR health networks))) AND ((emergency OR trauma OR EMS OR prehospital OR pre-hospital OR ambulance) OR ("Emergency Medicine"[Mesh] OR "Emergency Medical Services"[Mesh] OR

"Ambulances"[Mesh] OR "Emergency Treatment"[Mesh]))) NOT (COVID)) AND ((Africa OR African OR Algeria OR Angola OR Benin OR Botswana OR "Burkina Faso" OR Burundi OR "Cabo Verde" OR Cameroon OR Cameroun OR "Canary Islands" OR "Cape Verde" OR "Central African Republic" OR Chad OR Comoros OR Congo OR "Cote d'Ivoire" OR "Democratic Republic of Congo" OR Djibouti OR Egypt OR Eritrea OR eSwatini OR Ethiopia OR Gabon OR Gambia OR Ghana OR Guinea OR Guinea- Bissau OR "Ivory Coast" OR Jamahiriya OR Kenya OR Lesotho OR Liberia OR Libya OR Madagascar OR Malawi OR Mali OR Mauritania OR Mauritius OR Mayotte OR Morocco OR Mozambique OR Namibia OR Niger OR Nigeria OR Principe OR Reunion OR Rwanda OR "Saint Helena" OR "Sao Tome" OR Senegal OR Seychelles OR "Sierra Leone" OR Somalia OR "St Helena" OR Sudan OR Swaziland OR Tanzania OR Togo OR Tunisia OR Uganda OR "Western Sahara" OR Zaire OR Zambia OR Zimbabwe) OR ("Africa"[Mesh]))

Google Scholar:

STEMI AND “SYSTEMS OF CARE” AND AFRICA

First 10 pages screened for Inclusion.
