# Supplementary material for: ST-Elevation Myocardial Infarction Systems of Care in Africa: A Scoping Review
Source: Glob Heart. 2026 Feb 17;21(1):11. doi: 10.5334/gh.1524 (PMC12922679; doi:10.5334/gh.1524)
Supplement: Appendix B. — Extraction Template. [file gh-21-1-1524-s2.pdf]

Appendix B

Extraction Template

Study title	Author(s)	Origin	Setting	Aims/purpose	Methodology	Themes
Management of acute coronary syndromes in Maghreb countries: The ACCESS (ACute Coronary Events — a multinational Survey of current management Strategies) registry	Abdelhamid Moustaghfir, Mohand Haddak, Rachid Mechmeche	Algeria, Morocco and Tunisia	Facility based	We sought to describe the epidemiology, management, and clinical outcomes of patients hospitalized with acute coronary syndromes (ACS) in three countries in western North Africa	Prospective, multinational, observational registry	☐ Patient Related Challenges<br>☐ Healthcare Funding challenges<br>☐ Healthcare Facility Challenges
Profile of cardiac disease in Cameroon and impact on health care services.	Tantchou Tchoumi, Jacques Cabral, Butera, Gianfranco.	Cameroon	Facility based	The aim of the study was to investigate the preparedness of health care services for patients presenting with CVD in general and specifically, in St. Elizabeth catholic general hospital Shisong, cardiac centre.	prospective observational study	☐ Healthcare Funding challenges<br>☐ Healthcare worker Challenges
Population awareness of cardiovascular disease and its risk factors in Buea, Cameroon	Aminde, Leopold Ndemnge, Takah, Noah, Ngwasiri, Calypse, Noubiap, Jean Jacques, Tindong, Maxime, Dzudie, Anastase, Veerman, J. Lennert.	Cameroon	Facility based	We aimed to assess the population awareness (and associated factors) of CVD types and risk factors in Buea, Cameroon.	This was community-based cross-sectional study	☐ Patient Related Challenges
Barriers to the Implementation of Primary PCI in the Management of STEMI in Egypt.	Shaheen, Sameh, Helal, Ayman, Anan, Islam.	Egypt	Both	The aim of the present study is to explore the current practice in STEMI management in Egypt, to<br>assess the resources and capabilities of governmental hospitals, and to identify the most common gaps, barriers, and potential areas for improvement to widely provide PPCI in Egypt	face-to-face qualitative in-depth interviews	☐ Patient Related Challenges<br>☐ Healthcare Funding challenges<br>☐ EMS / prehospital Challenges<br>☐ QI/QA / Legislation / policies<br>☐ Healthcare Facility Challenges<br>☐ Technology Gaps<br>☐ Healthcare worker Challenges

Development of Primary Percutaneous Coronary Intervention as a National Reperfusion Strategy for Patients with ST-Elevation Myocardial Infarction and Assessment of Its Use in Egypt	Sobhy, Mohamed; Elshal, Ahmed; Ghanem, Noha; Hasan-Ali, Hosam; Farag, Nabil; Okasha, Nireen; Farag, El Sayed; Sadaka, Mohamed; Abo El Enein, Hisham; Salama, Sameh; Khamis, Hazem; Shokry, Khaled; Ragy, Hany; Elshorbagy, Amany; Mehanna, Radwa	Egypt	Facility based	This study aims to identify the mean time to primary PCI (door to balloon time) for STEMI patients and thus assess the percentage of primary PCI for STEMI patients and its success rate in Egypt. It also aims to evaluate different patterns of treatment for STEMI patients by the assessment of treatment modality rates for thrombolytic therapy or PCI (balloon dilation and/ or stenting)	Observational - A long-term prospective, cross-sectional, multicenter registry study	☐ Patient Related Challenges<br>☐ EMS / prehospital Challenges<br>☐ QI/QA / Legislation / policies<br>☐ Healthcare Facility Challenges<br>☐ Healthcare worker Challenges
Factors Affecting Symptom Onset to First-Medical-Contact in Egyptian STEMI Patients	BalbaaAmira, ; ElGuindy, Ahmed; Natarajan, Madhu; Schwalm, Jon-David	Egypt	N/A	to elucidate the factors affecting symptom onset to FMC from the patients and health system<br>perspective at The Aswan Heart Center (AHC)	mixed-methods observational study	☐ Patient Related Challenges<br>☐ EMS / prehospital Challenges<br>☐ QI/QA / Legislation / policies
Implementation of a Regional STEMI Network in North Cairo (Egypt): Impact on The Management and Outcome of STEMI Patients	Shaheen, Sameh M; Saleh, Atef K; Okasha, Nireen K; Abdalhamid, Mohammed A; Fakhry, Hany M; Guindy, Ramez R;	Egypt	Both	The aim of this study was to examine the feasibility and impact of establishing a regional STEMI network on the management and outcomes of STEMI patients in north Cairo	Prospective multicenter cross-sectional observational study	☐ Patient Related Challenges<br>☐ EMS / prehospital Challenges<br>☐ QI/QA / Legislation / policies<br>☐ Healthcare Facility Challenges<br>☐ Technology Gaps<br>☐ Healthcare worker Challenges
Stent for Life Initiative placed at the forefront in Egypt 2011	Sobhy, Mohamed, Sadaka, Mohamed, Okasha, Nireen, Farag, El Sayed, Saleh, Ayman, Ismail, Hussein, Seteiha, Mohamed El, Ragy, Hany, Hameed, Mohamed Abdel, Mehanna, Radwa.	Egypt	Both	The Stent for Life registry was launched to access the current situation of the Egyptian population presenting with STEMI, and to determine what were the barriers to providing patients with cardiac problems appropriate care	This registry was conducted at 14 centres covered all the Egyptian regions - Porspective	☐ Patient Related Challenges<br>☐ Healthcare Funding challenges<br>☐ Healthcare Facility Challenges

Before the door: Comparing factors affecting symptom onset to first medical contact for STEMI patients between a high and low-middle income country.	Balbaa, Amira, ElGuindy, Ahmed, Pericak, Dan, Natarajan, Madhu K, Schwalm, J D.	Egypt	Facility based	to elucidate the factors affecting symptom onset to FMC from the patients and health system perspective, comparing a newly developed regional cardiac center in a LMIC and a wellestablished cardiac center in a HIC.	This mixed-methods observational study. A review of the AHC STEMI registry and the HGH STEMI registry was conducted. A modified version of the Response to Systems Questionnaire (RSQ) that captures the six domains and basic demographic data was developed and reviewed by a panel of experts from both AHC and McMaster University/Population health Research Institute / Hamilton Health Sciences	☐ Patient Related Challenges<br>☐ EMS / prehospital Challenges<br>☐ Healthcare Facility Challenges
Management quality indicators and in-hospital mortality among acute coronary syndrome patients admitted to tertiary hospitals in Ethiopia: prospective observational study.	Fanta, Korinan ; Daba, Fekede Bekele ; Tegene, Elsah ; Melaku, Tsegaye ; Fekadu, Ginenus ; Chelkeba, Legese	Ethiopia	Facility based	this study aimed to assess the clinical profile, management quality indicators and clinical outcomes of patients with ACS admitted to two tertiary hospitals in Ethiopia.	prospective observational study.	☐ Patient Related Challenges<br>☐ Healthcare Funding challenges<br>☐ EMS / prehospital Challenges<br>☐ QI/QA / Legislation / policies<br>☐ Healthcare Facility Challenges<br>☐ Healthcare worker Challenges
Prevention and treatment of cardiovascular disease in Ethiopia: a cost-effectiveness analysis	Mieraf Taddesse Tolla, Ole Frithjof Norheim , Solomon Tessema Memirie , Senbeta Guteta Abdisa, Awel Ababulgu , Degu Jerene , Melanie Bertram , Kirsten Strand , Stéphane Verguet6 and Kjell Arne Johansson	Ethiopia	Neither	to assess cost-effectiveness of prevention and treatment of ischemic heart disease (IHD) and stroke in an Ethiopian setting	a generalized cost-effectiveness analysis	☐ Healthcare Funding challenges

Treatment Outcomes of Patients with Acute Coronary Syndrome Admitted to Tikur Anbessa Specialized Hospital, Addis Ababa, Ethiopia	Kassahun Bogale, Desalew Mekonnen, Teshome Nedi and Minyahil Alebachew Woldu	Ethiopia	Facility based	to assess the treatment outcome and associated factors for ACS in Tikur Anbessa Specialized Hospital (TASH	A retrospective cross-sectional study	☐ Patient Related Challenges<br>☐ Healthcare Funding challenges<br>☐ QI/QA / Legislation / policies<br>☐ Healthcare Facility Challenges
Factors Associated with the Extent of Coronary Artery Disease and the Attained Outcome of Percutaneous Coronary Intervention at Gesund Cardiac and Medical Center (GCMC), Addis Ababa, Ethiopia	Shashu, Bekele Alemayehu; Baru, Ararso	Ethiopia	Facility based	assessing factors associated with the extent of coronary artery disease and the attained outcomes in patients undergoing percutaneous coronary intervention in Ethiopia's Gesund Cardiac Medical and Center	single center retrospective observational study	☐ Patient Related Challenges
**Delays in Cardiovascular Emergency Responses in Africa: Health System Failures or Cultural Challenges?**	Asamoah, Kofi Tekyi; Doku, Alfred; Akumiah, Florence; Ampofo, Eugene; Duodu, Fiifi; Agyekum, Francis; Hafez, Mohammed; Akamah, Joseph; Ossei-Gerning, Nicholas; Baligeh, James; Russell, Walter; Agyemang, Charles	Ghana	Neither	Given the ravaging effects of these emergencies on families, communities, and the economy, what are the most appropriate measures to mitigate them?	Opinion piece	☐ Patient Related Challenges
Primary PCI in the management of STEMI in sub-Saharan Africa: insights from Abidjan Heart Institute catheterisation laboratory	Arnaud Ekou, Hermann Yao, Isabelle Kouamé, Rolande Yao Boni, Esther Ehouman, Roland N'Guetta	Ivory Coast	Facility based	The aim of this study was to report the results of primary PCI and outcomes in the catheterisation laboratory of the Abidjan Heart Institute	a cross-sectional, observational study	☐ Patient Related Challenges<br>☐ Healthcare Funding challenges<br>☐ EMS / prehospital Challenges<br>☐ Healthcare Facility Challenges
Medium and long-term follow-up after ST-segment elevation myocardial infarction in a sub-Saharan Africa population: a prospective cohort study.	Yao, Hermann; Ekou, Arnaud; Hadéou, Aurore; N'Djessan, Jean-Jacques; Kouamé, Isabelle; N'Guetta, Roland;	Ivory Coast	Facility based	We aimed to assess medium and long-term prognosis in patients with STEMI admitted to Abidjan Heart Institute	A prospective, single centre study	☐ Patient Related Challenges<br>☐ Healthcare Funding challenges<br>☐ Healthcare Facility Challenges

Optimizing the management of acute coronary syndromes in sub-Saharan Africa: A statement from the AFRICARDIO 2015 Consensus Team	Kakou-Guikahue, Maurice, N'Guetta, Roland, Anzouan-Kacou, Jean Baptiste, Kramoh, Euloge, N'Dori, Raymond, Ba, Serigne Abdou, Diao, Maboury, Sarr, Moustapha, Kane, Abdoul, Kane, Adama, Damorou, Findide, Balde, Dadhi, Diarra, Mamadou Bocary, Djiddou, Mohamed, Kimbally-Kaki, Gisèle, Zabsonre, Patrice, Toure, Ibrahim Ali, Houénassi, Martin, Gamra, Habib, Chajai, Bachir, Gerardin, Benoit, Pillière, Rémy, Aubry, Pierre, Iliou, Marie Christine, Isnard, Richard, Leprince, Pascal, Cottin, Yves, Bertrand, Edmond, Juillière, Yves, Monsuez, Jean Jacques.	Ivory Coast	Both	To propose a consensus statement to optimize management of ACS in sub-Saharan Africa on the basis of realistic considerations.	Conference consensus statement	☐ Patient Related Challenges<br>☐ Healthcare Funding challenges<br>☐ EMS / prehospital Challenges<br>☐ Healthcare Facility Challenges
Tele-ECG improves diagnosis of acute coronary syndrome and ST-elevation myocardial infarction in Côte d'Ivoire	Diby, K. F. Gnaba, A. Ouattara, P. Ayegnon, G. Coulibaly, A. Tro, G. Dakoi, S. A. Sall, F. Adoubi, A. N'guessan, K. E. Ehua, S. F. Ohannessian, R. Moulin, T.	Ivory Coast	Facility based	To define the prevalence of STEMI and to describe the characteristics of patients diagnosed with STEMI within the tele-electrocardiogram (ECG) network in Côte d'Ivoire.	Retrospective Data Collection	☐ Technology Challenges
A prospective review of acute coronary syndromes in an urban hospital in sub-Saharan Africa	JAY SHAVADIA, GERALD YONGA, HARUN OTIENO	Kenya	Facility based	We set out to define the demographics, presentation and outcomes of patients admitted with an acute coronary syndrome (ACS) at the Aga Khan University Hospital, Nairobi (AKUHN	A prospective survey	☐ Patient Related Challenges<br>☐ Helthcare Funding challenges<br>☐ EMS / prehospital Challenges<br>☐ QI/QA / Legislation / policies<br>☐ Healthcare Facility Challenges<br>☐ Technology Gaps<br>☐ Healthcare worker Challenges

Outcomes in patients with acute coronary syndrome in a referral hospital in sub-Saharan Africa	Varwani, Mohamed Hasham, Jeilan, Mohamed, Ngunga, Mzee, Barasa, Anders	Kenya	Facility based	To determine the in-hospital and long-term (30-day and one-year) mortality rates of ACS patients treated at the Aga Khan University Hospital, Nairobi (AKUHN). Secondary objectives were to determine the rate of in-hospital non-fatal events, specifically heart failure, recurrent myocardial infarction (MI), need for repeat revascularisation, stroke and major bleeding, and to determine the rate of rehospitalisation in the first year owing to major adverse events (recurrent MI, stroke and major bleeding).	a cross-sectional, retrospective review	☐ Patient Related Challenges<br>☐ QI/QA / Legislation / policies<br>☐ Healthcare Facility Challenges<br>☐ Technology Gaps
Presentation, management and outcomes of acute coronary syndrome: a registry study from Kenyatta National Hospital in Nairobi, Kenya	Ehete Bahiru, Tecla Temu, Bernard Gitura, Carey Farquhar, Mark D Huffman, Frederick Bukachi	Kenya	Facility based	We sought to create an ACS registry at Kenyatta National Hospital to evaluate the presentation, management and outcomes of ACS patients	a retrospective chart review	☐ Patient Related Challenges<br>☐ QI/QA / Legislation / policies<br>☐ Healthcare Facility Challenges<br>☐ Technology Gaps
Understanding of and perceptions towards cardiovascular diseases and their risk factors: a qualitative study among residents of urban informal settings in Nairobi	Murunga, WekesahFrederick, Kyobutungi, Catherine, Grobbee, Diederick E, Klipstein-Grobusch, Kerstin.	Kenya	out-of-hospital	In this study conducted among the residents of Nairobi slums, where more than half of the urban dwellers of the city live, we investigated the understanding of and perceptions towards CVD and their risk factors and how the understanding and perception affected actions taken by individuals to prevent CVD and in seeking care and adhering to treatment	phenomenological qualitative study, focus group discussions	☐ Patient Related Challenges
Acute Coronary Syndrome patterns in the Young: risk factor profile and in-hospital outcome in a tertiary referral hospital in Kenya	Varwani, Mohamed, Ngunga, Mzee, Msunza, Miriam, Mohamed, Jeilan	Kenya	Facility based	The study aimed to describe the characteristics of young individuals hospitalized with ACS and report on in-hospital outcomes.	This single-center retrospective study	☐ Patient Related Challenges
Evaluation of risk factors in acute myocardial infarction patients admitted to the coronary care unit, Tripoli Medical Centre, Libya	Abduelkarem, A. R., El-Shareif, H. J., Sharif, S. I.	Libya	Facility based	The aim of this study was to provide an overview of the risk factors for acute myocardial infarction in patients attending Tripoli Medical Centre, Libya.	Review of Case Records	☐ Patient Related Challenges<br>☐ Healthcare Facility Challenges

Registry for Acute Coronary Events in Nigeria (RACE-Nigeria): Clinical Characterization, Management, and Outcome	Isezuo, Simeon, Sani, Mahmoud Umar, Talle, Abdullahi, Johnson, Adeyemi, Adeoye, Abiodun Moshood, Ulgen, Mehmet S., Mbakwem, Amam, Ogah, Okechukwu, Edafe, Emmanuel, Kolo, Philip, Nagabea, Murtala, Adebayo, Rasaaq, Nwafor, Eze, Daniel, Folasade, Zagga, Muiyawa, Umar, Hayatu, Oboirien, Isa, Sulaiman, Balarabe A., Abdullahi, Umar, Mijinyawa, Muhammad Sani, Buba, Farouk, Aje, Akinyemi, Okolie, Henry, Shehu, Muhammad Nazir, Adamu, Umar, Olusegun-Joseph, Akinsanya, Familoni, Ranti, Chibuzor, Nwuriku, Olunuga, Taiwo Olabisi, Ejim, Emmanuel, Olaide, Awodu Rasheed, Ojji, Dike, Sanni, Bushra, Ajuluchukwu, Jane N., Balogun, Michael O., Omotoso, Ayodele B., Ajit, Mullasari, Falase, Ayodele	Nigeria	Facility based	The Registry for Acute Coronary Events in Nigeria (RACE-Nigeria) is aimed at determining the incidence, peculiarities in the characteristics, management, and all-cause mortality of ACS	A prospective, observational, and multicentered	☐ Patient Related Challenges<br>☐ Healthcare Funding challenges<br>☐ EMS / prehospital Challenges<br>☐ QI/QA / Legislation / policies<br>☐ Healthcare Facility Challenges
**Door-to-Balloon Time and Mortality Among Patients Undergoing Primary PCI, Challenges and Experience from Somalia's Largest PCI Center**	Omar Hassan, Mohamed Abdirahman Ahmed, Said Sheikh Hassan, Mohamed Köprülü, Diyar	Somalia	Facility based	Angiographical records were analyzed to assess diseased vessels, contrast used, balloon time, access site, stents used, radiation dose, and other procedures during primary PCI	Retrospective analysis	☐ Healthcare Facility Challenges

Management of acute coronary syndrome in South Africa: insights from the ACCESS (Acute Coronary Events – a Multinational Survey of Current Management Strategies) registry	COLIN SCHAMROTH, ACCESS South Africa investigators	South-Africa	Facility based	to gain insights into the descriptive epidemiology, current practice patterns, and one-year outcomes of patients hospitalised with acute coronary syndrome (ACS), whether this be unstable angina (UA)/non-STsegment elevation acute coronary syndrome (NSTE-ACS) or ST-segment elevation myocardial infarction (STEMI), in developing countries	prospective, observational registry	☐ Patient Related Challenges<br>☐ Healthcare Funding challenges<br>☐ EMS / prehospital Challenges<br>☐ QI/QA / Legislation / policies<br>☐ Healthcare Facility Challenges
Evaluating the time interval from diagnosis to fibrinolysis at centres in the drainage area of Tygerberg Hospital, Cape Town, South Africa	BeyersB, D; Doubell, A F; Griffiths, B; Jalavu, T	South-Africa	Facility based	To determine the median time interval between diagnosis and fibrinolysis in patients presenting to centres within the drainage area of Tygerberg Hospital, Cape Town, SA,	retrospective medical record review	☐ Patient Related Challenges<br>☐ QI/QA / Legislation / policies<br>☐ Healthcare Facility Challenges<br>☐ Healthcare worker Challenges
Non-ST elevation myocardial infarction (NSTEMI) in three hospital settings in South Africa: does geography influence management and outcome? A retrospective cohort study	Moses, Jane; Doubell, Anton F; Herbst, Philip G; Klusmann, Karl J C; Weich, Hellmuth S V H	South-Africa	Facility based	This study aimed to determine whether the management of an NSTEMI differs depending on the hospital to which the patient presents (patients presenting to secondary hospitals being less likely to receive early invasive management), and if so, whether this is a consequence of geographical remoteness or level of care, and how this influences outcome.	retrospective cohort study	☐ Healthcare worker Challenges
The 12-month period prevalence and cardiac manifestations of HIV in patients with acute coronary syndrome at a tertiary hospital in Cape Town, South Africa: a retrospective cross-sectional study.	Pennefather, Camilla; Esterhuizen, Tonya; Doubell, Anton; Decloedt, Eric H;	South-Africa	Facility based	The objective of this study was to determine the 12-month period prevalence of HIV in patients with ACS and to compare the risk-factor profile, ACS presentation and management between HIV-positive and HIV-negative adults	We conducted a retrospective review	☐ Patient Related Challenges
Time to fibrinolytics for acute myocardial infarction: Reasons for delays at Steve Biko Academic Hospital, Pretoria, South Africa	Meel, R, Gonçalves, R.	South-Africa	Facility based	To establish the proportion of STEMI patients receiving fibrinolytic agents at Steve Biko Academic Hospital (SBAH), Pretoria, SA, identify any delays to receiving fibrinolytic agents, and uncover reasons for those delays	prospective, observational study	☐ Patient Related Challenges<br>☐ Healthcare Funding challenges<br>☐ EMS / prehospital Challenges<br>☐ QI/QA / Legislation / policies<br>☐ Healthcare Facility Challenges<br>☐ Technology Gaps<br>☐ Healthcare worker Challenges

The proportion of South Africans living within 60 and 120 minutes of a percutaneous coronary intervention facility	Willem Stassen, Lee Wallis, Craig Vincent-Lambert, Maaret Castren, Lisa Kurland	South-Africa	Facility based	The aim of this study was to determine the proportion of South Africans living within 60 and 120 minutes of a PCI facility.	PCI facility and population data were subjected to proximity analysis to determine the average drive times from municipal ward centroids to PCI facilities for each province in South Africa - mapping	☐ Healthcare Funding challenges<br>☐ Healthcare Facility Challenges
The incidence and outcomes of high-risk acute coronary syndromes in the Western Cape Province of South Africa: a prospective cohort study	Cilliers, J D ; Joubert, L ; Beyers, B ; Ngarande, E ; Herbst, P ; Doubell, A ; Pecoraro, A	South-Africa	Facility based	To describe the incidence of ST-elevation myocardial infarction (STEMI) and high-risk non-ST-elevation ACS (HR-NSTEACS) in the TBH referral network, describe the in-hospital and 30-day mortality of these patients, and identify important high-risk population characteristics	prospective cohort study	☐ Patient Related Challenges<br>☐ QI/QA / Legislation / policies<br>☐ Healthcare Facility Challenges
The application of optimisation modelling and geospatial analysis to propose a coronary care network model for patients with ST-elevation myocardial infarction.	Stassen, Willem, Olsson, Leif, Kurland, Lisa.	South-Africa	Both	the aim of this study was to propose the optimal reperfusion strategy in a coronary care network model for patients who present with STEMI	This study applied geospatial analysis with network optimisation modelling, to determine which strategy (thrombolysis or PCI) is most appropriate for patients presenting within each of the municipal wards of the North West province. - mapping	☐ Healthcare Facility Challenges<br>☐ Technology Gaps
Profile and management of acute coronary syndromes at primary- and secondary-level healthcare facilities in Cape Town	Uys, F., Beeton, A. T., van der Walt, S., Lamprecht, M., Verryn, M., Vallie, Y., Stokes, D., Millar, R. S., Viljoen, C. A.	South-Africa	Facility based	to describe the profile, clinical presentation and management of patients with ACS treated at primaryand secondary-level healthcare facilities in Cape Town, South Africa.	We conducted a retrospective folder review	☐ Patient Related Challenges<br>☐ EMS / prehospital Challenges<br>☐ Healthcare Facility Challenges<br>☐ Healthcare worker Challenges
Door-to-needle time for administration of fibrinolytics in acute myocardial infarction in Cape Town	Maharaj, Roshen C., Geduld, Heike, Wallis, Lee A.	South-Africa	Facility based	To determine the current door-to-needle time for the administration of fibrinolytics for acute myocardial infarction (AMI) in emergency centres (ECs) at three hospitals in Cape Town	A retrospective review of case notes	☐ Patient Related Challenges<br>☐ EMS / prehospital Challenges<br>☐ QI/QA / Legislation / policies<br>☐ Healthcare Facility Challenges

Chart review of acute myocardial infarction at a district hospital in KwaZulu-Natal, South Africa	Chetty, Roland, Ross, Andrew.	South-Africa	Facility based	The aim of the study was to determine the profile and management of patients admitted with ischaemic chest pain.	The design was retrospective and descriptive	☐ Patient Related Challenges<br>☐ Healthcare Facility Challenges<br>☐ Healthcare worker Challenges
Barriers to the implementation of prehospital thrombolysis in the treatment of ST-segment elevation myocardial infarction in South Africa: An exploratory inquiry.	Lynch, Andrew, Sobuwa, Simpiwe, Castle, Nicholas	South-Africa	out-of-hospital	The current study aimed to explore, through a qualitative inquiry, barriers to PHT for the treatment of myocardial infarction within a South African context.	A qualitative single-case study design was used where a series of semi-structured interviews were conducted involving purposefully selected participants	☐ Healthcare Funding challenges<br>☐ EMS / prehospital Challenges<br>☐ QI/QA / Legislation / policies<br>☐ Healthcare worker Challenges
Barriers and facilitators to implementing coronary care networks in South Africa: a qualitative study	Stassen, Willem, Kurland, Lisa, Wallis, Lee, Castren, Maaret, Vincent-Lambert, Craig	South-Africa	Both	To determine the current perceived state of CCNs, to determine the barriers to optimising CCNs and to suggest facilitators to optimising CCNs within the South African context.	A qualitative descriptive approach was employed, by performing two structured in-depth and two focus group interviews	☐ Patient Related Challenges<br>☐ Healthcare Funding challenges<br>☐ EMS / prehospital Challenges<br>☐ QI/QA / Legislation / policies<br>☐ Healthcare Facility Challenges<br>☐ Technology Gaps<br>☐ Healthcare worker Challenges
Acute myocardial infarction at a district hospital in KwaZulu-Natal – Management and outcomes	Badat Zakariya, Rangiah Selvandran.	South-Africa	Facility based	This study analysed the management of patients presenting with STEMI and NSTEMI as well as the outcomes in a district-level resource-limited environment with no PCI or on-site cardiology service.	descriptive cross-sectional study	☐ Patient Related Challenges<br>☐ EMS / prehospital Challenges<br>☐ QI/QA / Legislation / policies<br>☐ Healthcare Facility Challenges<br>☐ Healthcare worker Challenges
Time to thrombolysis and factors contributing to delays in patients presenting with ST-elevation myocardial infarction at Chris Hani Baragwanath Academic Hospital, Johannesburg, South Africa	Tickley, I, van Blydenstein, S A, Meel, R.	South-Africa	Both	The primary objective of this study was to elucidate the time to thrombolysis and reasons for delays in administration or nonadministration of thrombolytic agents to patients with STEMI presenting to CHBAH. Secondary objectives were to determine the demographics of patients presenting with STEMI, and their echocardiogram and coronary angiogram findings	This single-centre prospective observational study	☐ Patient Related Challenges<br>☐ Healthcare Funding challenges<br>☐ EMS / prehospital Challenges<br>☐ QI/QA / Legislation / policies<br>☐ Healthcare Facility Challenges<br>☐ Technology Gaps<br>☐ Healthcare worker Challenges

Referral pathways for reperfusion of STEMI- developing strategies for appropriate intervention: the SA heart STEMI early intervention project	Adriaan Snyders and Rhena Delport	South-Africa	Both	to establish the current time intervals present in the referral pathways to percutaneous coronary intervention (PCI) facilities in the Tshwane Metropole and to further identify the barriers to appropriate management of STEMI.	A cross-sectional observational study	☐ Patient Related Challenges<br>☐ Healthcare Funding challenges<br>☐ EMS / prehospital Challenges<br>☐ Healthcare Facility Challenges<br>☐ Technology Gaps
STEMI early reperfusion programme: cardiology	Snyders, Adriaan.	South-Africa	Facility based	Article discussing the South African Society of Cardiovascular Intervention (SASCI) ST Segment Elevation Myocardial Infarction (STEMI) Early Reperfusion Programme's call to action to implementation in Gauteng and the rest of South Arica	Editorial	☐ Patient Related Challenges<br>☐ Healthcare Funding challenges<br>☐ QI/QA / Legislation / policies<br>☐ Healthcare Facility Challenges<br>☐ Healthcare worker Challenges
Revealing the value of geospatial information with isochrone maps for improving the management of heart attacks in South Africa	Coetzee, Serena, Snyman, Lourens, Delport, Rhena.	South-Africa	Facility based	In this essay we describe a map, recently prepared for the South African Heart Association STEMI SA Early Intervention Initiative, that envisages to improve systems of care to afford timely and appropriate management of ST-elevation Myocardial Infarction (STEMI) heart attacks	The map shows geographic access to public cathlabs based on a maximum drive-time threshold of two hours, which is sub-divided into thirty-minute intervals. Maptitude was utilised for the isochrone modelling and map visualization. the data package for South Africa, shipped with the Maptitude software, was used together with OpenStreetMap placenames - mapping	☐ Healthcare Facility Challenges<br>☐ Technology Gaps
Coronary care networks in the resource-limited setting: systems of care in South Africa	Stassen, Willem.	South-Africa	Facility based	Determine the amount and location of PCI-facilities in South Africa and to relate coverage to population; and access in relation to socio-economic status for each South African province	cross-sectional	☐ Healthcare Facility Challenges

Gender inequality in acute coronary syndrome patients at Omdurman Teaching Hospital, Sudan	Mirghani, Hyder O; Elnour, Mohammed A; Taha, Akasha M; Elbadawi, Abdulateef S;	Sudan	Facility based	To assess gender differences in presentation, management, and outcomes of acute coronary syndrome in Sudan.	cross-sectional descriptive longitudinal study	☐ Patient Related Challenges
Knowledge, attitudes, and preventative practices regarding ischemic heart disease among emergency department patients in northern Tanzania.	Hertz, J T; Sakita, F M; Manavalan, P; Mmbaga, B T; Thielman, N M; Staton, C A	Tanzania	Facility based	To increase understanding of knowledge, attitudes, and preventative practices regarding ischemic heart disease (IHD) in sub-Saharan Africa in order to develop patient-centered interventions to improve care and outcomes	prospective observational study	☐ Patient Related Challenges
Management of patients with acute STelevation myocardial infarction: Results of the FAST-MI Tunisia Registry	Faouzi AddadID , Abdallah Mahdhaoui , Jeridi Gouider , Essia Boughzela, Samir Kamoun4, Mohamed Rachid Boujnah5, Habib Haouala6 , Habib Gamra, Faouzi Maatouk , Ali Ben Khalfallah , Salem Kachboura , Hedi Baccar, Nejeh Ben Halima, Ali Guesmi, Khaled Sayahi, Wissem Sdiri, Ali Neji, Ahmed Bouakez, Sami Milouchi, Kais Battikh, Yves Jullieres, Nicolas Danchin, Jean Jacques Monsuez, Genevieve Mulak, Albert Hagege, Vincent Bataille, Rafik Chettaoui, Mohamed Sami Mourali,	Tunisia	Both	we sought to analyse the demographic and the clinical characteristics as well as the modalities of myocardial reperfusion employed in STEMI patients enrolled into the FAST-MI Tunisia registry comparing the management strategies between university (generally with cath lab) and regional hospitals (without cath lab), and investigating the independent predictors of in-hospital mortality.	prospectively	☐ Patient Related Challenges<br>☐ EMS / prehospital Challenges<br>☐ QI/QA / Legislation / policies<br>☐ Healthcare Facility Challenges

Management of acute coronary syndrome in emergency departments: a cross sectional multicenter study (Tunisia)	Sriha Belguith, Asma; Beltaief, Kaouthar; Msolli, Mohamed Amine; Bouida, Wahid; Abroug, Hela; Ben Fredj, Manel; Zemni, Imen; Grissa, Mohamed Habib; Boubaker, Hamdi; Hsairi, Mohamed; Nouira, Samir;	Tunisia	Facility based	We aimed to describe diagnosed acute coronary syndrome (ACS) and its care management and outcomes in emergency departments (EDs) and to determine related cardiovascular risk factors (CVRFs).	a multicenter cross-sectional study. Data were prospectively collected	☐ Patient Related Challenges<br>☐ EMS / prehospital Challenges<br>☐ QI/QA / Legislation / policies<br>☐ Healthcare worker Challenges
Predictors of acute coronary syndrome in pre hospital patients with chest pain	Saida Zelfani, Selim Boudiche, Héla Manai, M Sami Mourali, Mounir Daghfous	Tunisia	out-of-hospital	The objective of our study was to identify predictive factors of STEMI in patients with acute chest pain, in the Emergency Care System of the North Est (SAMU 01) of Tunisia.	a prospective observational study, through telephonic interviews.	☐ Patient Related Challenges<br>☐ Healthcare Facility Challenges<br>☐ Healthcare worker Challenges
Delay of Fibrinolysis in ST-Elevation Myocardial Infarction: Results of an Investigation Conducted in a Single Center in Sousse Tunisia	Bouraoui, Hatem, Trimeche, Besma, Hajri, Samia Ernez, Mahdhaoui, Abdallah, Romdhane, Meriem Ben, Jeridi, Gouider.	Tunisia	Facility based	The aim of our study was to assess the delay of fibrinolysis in ST elevation myocardial infarction (STEMI) in our region and to identify characteristics associated with prolonged delay	prospective cohort study	☐ EMS / prehospital Challenges<br>☐ QI/QA / Legislation / policies<br>☐ Healthcare Facility Challenges<br>☐ Healthcare worker Challenges
STEMI care in the elderly: Does under-treatment reflect appropriate clinical judgment or therapeutic nihilism?	Gupta, Tanush.	Tunisia	Facility based	we sought to determine in-hospital outcomes for elderly patients presenting with STEMI in a Tunisian center and to study factors related to in-hospital death in this population	Single Centre retrospective observational study	☐ Patient Related Challenges<br>☐ Healthcare Facility Challenges
